# Fusionless All-Pedicle Screws for Posterior Deformity Correction in AIS Immature Patients Permit the Restoration of Normal Vertebral Morphology and Removal of the Instrumentation Once Bone Maturity Is Reached

**DOI:** 10.3390/jcm12062408

**Published:** 2023-03-21

**Authors:** Jesús Burgos, Gonzalo Mariscal, Luis Miguel Antón-Rodrigálvarez, Ignacio Sanpera, Eduardo Hevia, Vicente García, Carlos Barrios

**Affiliations:** 1 Spine Unit, Hospital Viamed Fuensanta, 28027 Madrid, Spain; 2 School of Doctorate, Valencia Catholic University, 46001 Valencia, Spain; gonzalo.mariscal@mail.ucv.es; 3 Pediatric Orthopedics, Ramon y Cajal Hospital, 28034 Madrid, Spain; 4 Pediatric Orthopedics, Hospital Son Espases, 07198 Palma de Mallorca, Spain; 5 Spine Unit, Hopsital La Fraternidad-Muprespa, 28036 Madrid, Spain; 6 Sección de Cirugía de Columna, Hospital Universitario Araba, 01009 Vitoria, Spain; 7 Institute for Research on Musculoskeletal Disorders, Valencia Catholic University, 46001 Valencia, Spain

**Keywords:** fusionless technique, posterior instrumentation, adolescent idiopathic scoliosis, vertebral modulation, pedicle screw stabilization, coronal wedging ratio

## Abstract

The aim of this study was to report the restoration of normal vertebral morphology and the absence of curve progression after the removal of instrumentation in AIS patients that underwent posterior correction of the deformity by a common all-screws construct without fusion. A series of 36 AIS immature patients (Risser 3 or less) were included in the study. Instrumentation was removed once the maturity stage was complete (Risser 5). The curve correction was assessed pre- and postoperatively, before instrumentation removal, directly post-removal, and more than two years after instrumentation was removed. Epiphyseal vertebral growth modulation was assessed by the coronal wedging ratio (WR) at the apical level of the main curve (MC). The mean preoperative coronal Cobb was corrected from 53.7° ± 7.5 to 5.5° ± 7.5° (89.7%) at the immediate postop. After implant removal (31.0 ± 5.8 months), the MC was 13.1°. T5–T12 kyphosis showed significant improvement from 19.0° before curve correction to 27.1° after implant removal (*p* < 0.05). Before surgery, the WR was 0.71 ± 0.06, and after removal, 0.98 ± 0.08 (*p* < 0.001). At the end of the follow-up, the mean sagittal range of motion (ROM) of the T12-S1 segment was 51.2 ± 21.0°. The SRS-22 scores improved from 3.31 ± 0.25 preoperatively to 3.68 ± 0.25 at the final assessment (*p* < 0.001). In conclusion, a fusionless posterior approach using common all-pedicle screws correctly constructed satisfactory scoliotic main curves and permitted the removal of instrumentation once bone maturity was reached. The final correction was highly satisfactory, and an acceptable ROM of the previously lower instrumented segments was observed.

## 1. Introduction

Current techniques for the surgical correction of adolescent idiopathic scoliosis are focused on correcting vertebral deformity and preventing its progression. Two surgical procedures have been developed to achieve these goals. The first involves the instrumental correction and fusion of the affected vertebral segment. The second, based on vertebral modulation, applies corrective forces to modulate the growth of vertebral epiphyseal plates by means of implants [1].

Vertebral growth modulation is based on the Hueter–Volkmann Law [2], according to which the application of compressive forces causes the slowing of physeal bone growth, and opposite distraction forces increase bone growth. These forces can be applied to the scoliotic spine with the aim of restoring normal vertebral morphology [3,4].

There are two types of vertebral modulation, which can be differentiated by the surgical approach and where the implants are placed to produce the corrective forces. In the first, the approach and the corrective forces are applied in the anterior part of the vertebrae, placing the implants in the convexity of the vertebral bodies. For this type of anterior modulation, there are two methods: vertebral body stapling [5] and tethering [6]. In both techniques, primarily compressive forces are applied to decrease the vertebral growth of the convexity. In the second type of vertebral modulation, the corrective forces are applied posteriorly by implants located in the vertebral pedicles. This method has been experimentally demonstrated to produce vertebral modulation [7,8,9]. There is also recent clinical evidence showing vertebral modulation using distracting forces through pedicle screws located at the ends of the concavity [10].

In this study, we report the restoration of normal vertebral morphology and the absence of curve progression after the removal of instrumentation in AIS patients who underwent posterior correction of the deformity by a common bilateral all-screws construct without fusion. The initial correction surgery was performed when the disease was immature (Risser 0–3). Once bone maturation was achieved, the vertebral instrumentation was removed to restore the mobility of all vertebral segments, particularly at the thoracolumbar junction and lumbar segments.

## 2. Materials and Methods

### 2.1. Design

A retrospective study was carried out on a longitudinal series of patients with adolescent idiopathic scoliosis treated surgically using a fusionless posterior vertebral modulation system, as described below. All study participants and their parents were informed about the surgical procedure and provided written consent. The work was conducted following the European recommendations of good clinical practice and the principles of the Helsinki declaration of the World Medical Assembly, revised in 2013 for clinical studies in humans. The treatment applied to the patients was performed following the deontological standards proposed by the Spanish Spine Society. This study was approved by the ethical committee of our institution (UCV/2021-2022/109).

### 2.2. Participants

A total of thirty-six skeletally immature patients with adolescent idiopathic scoliosis were included in the study. The inclusion criteria were adolescents with idiopathic scoliosis, skeletally immature (Risser’s sign 3 or lower), with flexible curves greater than 45° Cobb. Patients with open tri-radiate cartilage, those with severe rigid curves, and cases that did not meet all the parameters analyzed in this investigation were excluded from this study.

### 2.3. Intervention

In all cases, a conventional surgical technique was used for scoliosis correction via a posterior approach, placing pedicle screws in necessary quantities in the area to be instrumented. The instrumentation was introduced to end proximally and distally in the horizontal vertebrae or with an angulation of less than 10° with respect to the horizontal in the anteroposterior teleradiograph. The study included only scoliosis that was corrected to less than 10° Cobb in the immediate postoperative radiography, or hypercorrected to less than 10° on the opposite side.

The same pedicle-screw technique was applied consistently to achieve maximum correction in all three planes. This technique is well-validated and has been internationally standardized, allowing individual correction of each of the three planes with minimal surgical maneuvers. The following is a brief description of the technique:

Two senior surgeons performed the surgery, with the patient under general anesthesia in a prone position and with neurophysiological monitoring. The levels to be instrumented were exposed in a conventional manner through a posterior approach without injuring the vertebral ligamentous elements or the facet joint capsules. Titanium pedicle screws were placed bilaterally from distal to proximal at all levels to be instrumented, using the free-hand technique. Neurophysiological control of the screw trajectory was always performed prior to insertion and t-EMG screw stimulation was applied once positioned.

In all cases, transverse hooks were placed on both sides at the proximal level of instrumentation to avoid tension forces at the transitional area, as many practitioners do. Two long tulip polyaxial screws were also placed at the convexity proximal level, and two long tulip polyaxial screws were inserted at the distal level. In the middle of the convexity, uniplanar cephalocaudal screws were alternated with long polyaxial screws to facilitate the insertion of the pre-contoured rod without complete tapping of the rod into the tulips. In more flexible curves, it was possible to use more polyaxial screws with a longer tulip which facilitated the insertion of the pre-contoured rod. In the concavity of the curves, long polyaxial screws were used at all levels.

In all cases, cobalt–chrome rods were used. The rods were molded only in the sagittal plane and asymmetrically, giving greater thoracic kyphosis and greater lumbar lordosis to the thoracic concavity rod and less kyphosis and lordosis to the rod in the convexity. Lordosis was applied to the concavity rod just two centimeters from the proximal end of the rod in order to facilitate placement into the hooks and proximal implants, avoiding pull-outs at this level. The concavity and convexity rods were bent asymmetrically, giving more height to the concave rod. Removal of the instrumentation was accomplished when at least Risser IV was achieved, ensuring that all patients were skeletally mature at the time of implant removal.

### 2.4. Outcomes

The following parameters were analyzed in each patient: age, gender, weight, height, Tanner stage, menarche before surgery, and age at menarche. Anteroposterior and lateral teleradiographs were analyzed preoperatively, immediately postoperatively after correction, prior to the instrumentation removal of the implants, immediately after instrumentation removal, and a minimum of two years after implant removal. The following parameters were obtained in these teleradiographs: Risser stage, side of scoliosis, Lenke type, coronal Cobb degrees of curves, limiting vertebrae, Perdriolle degrees (preoperative and final radiographs only), and coronal vertebral imbalance in the anteroposterior X-rays.

**Figure 1:**
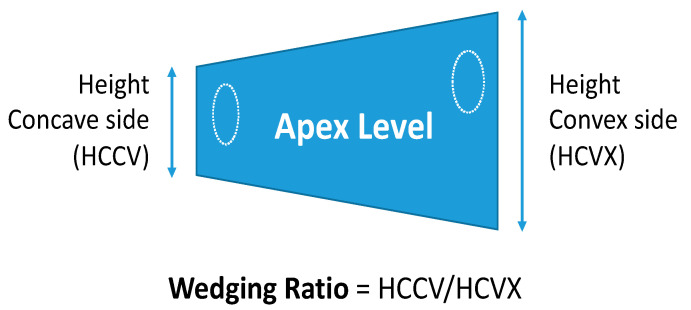
The wedging ratio (WR) represents the effect on the vertebral morphology of the growth of the concave epiphyseal plate at the apical vertebra, and is calculated by the ratio between the height of the concavity and convexity.

The restoration of the vertebral morphology was quantified at the apical vertebra in the preoperative and final anteroposterior radiographs by measuring the wedging ratio (WR): the relation between the height of the concavity and convexity (Figure 1).

In the anteroposterior X-rays, the proximal and distal instrumented vertebrae and the number of instrumented pedicles were recorded in each case. In the lateral X-rays, T2–T12 thoracic kyphosis, T12-S1 lumbar lordosis, and sagittal imbalance were observed. Two years after removal of the instrumentation, lateral radiographs of the lumbosacral spine in maximum flexion and extension were taken in the standing position, with the objective of analyzing the mobility of the lumbar segments that were included in the posterior stabilization. Thoracic range of motion (T1–T12) after the removal of implants was not considered, due to its limited physiologic value in AIS patients before surgery (1.1 ± 1.5° Cobb) and low impact on the whole sagittal ROM of the spine (4.8%) [11].

SRS-22 questionnaires were assessed preoperatively and at the end of the study. The SRS-22 was administered by the medical team responsible for the treatment of the patients at the time of check-up at the outpatient clinic. Similar instructions were given to all the participants.

### 2.5. Statistical Analysis

The entire cohort was described in terms of demographics and surgical metrics using standard deviation and means. The evolution of surgical correction throughout the postoperative period, especially after the removal of instrumentation, was analyzed using the Wilcoxon test. Two groups of curves, including thoracic and thoracolumbar/lumbar, were compared in order to identify differences in the final results. Patients exhibiting Risser 0–1 were compared with those showing Risser 2–3. Statistical analysis was performed with the SPSS 24.0 package (IBM, Chicago, IL, USA). A value of *p* < 0.05 was considered statistically significant.

## 3. Results

### 3.1. Baseline Data

**Figure 2:**
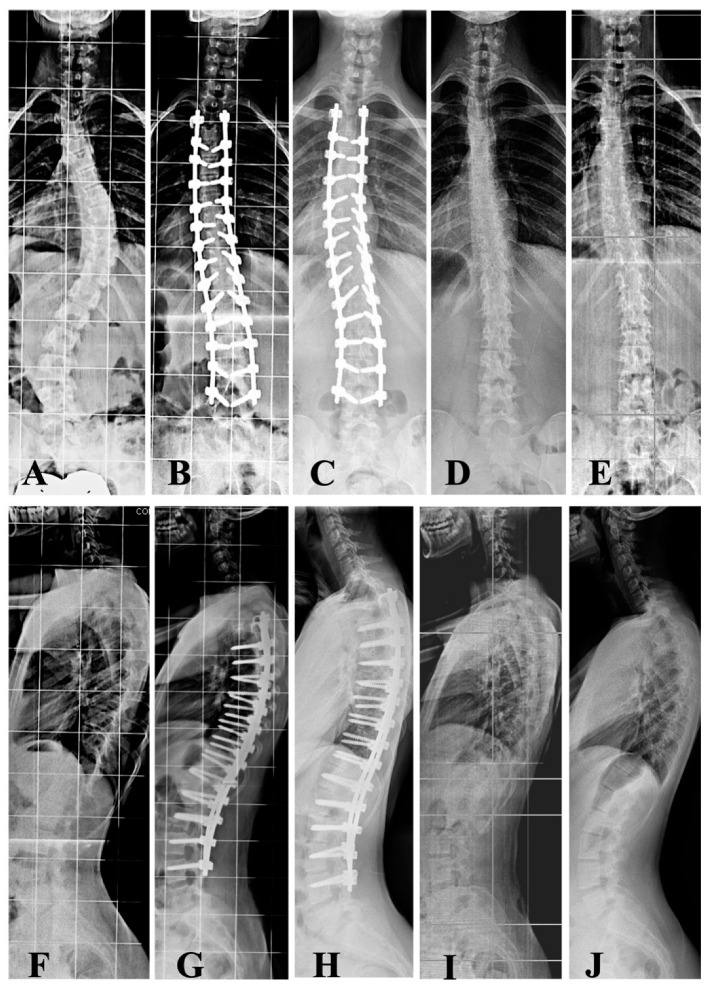
A 13-year-and-2-month-old girl with Lenke type 1BN AIS (50°/36°) with Risser 1 (**A**) and thoracic hypokyphosis of 13° (**F**), was surgically treated by a posterior approach involving T3-L4 instrumentation without fusion with slight hypercorrection of both curves (**B**). Three years and two months after the initial surgical intervention and just before the removal of instrumentation, the main and compensatory curves remained unchanged towards the opposite side of the initial curves (**C**). The radiograph after removal of the vertebral implants shows minimal residual scoliosis towards the opposite side of the curves that existed before surgical treatment (**D**). These minimal scoliotic curves remained unchanged on the last radiograph, taken two years after the removal of the instrumentation (**E**). Concerning the sagittal plane, the postoperative radiograph after surgical correction demonstrated an increase in previous hypokyphosis (**G**), which remained unchanged in the radiograph prior to removal of the instrumentation (**H**), after its removal (**I**), and in the last X-ray check-up two years after instrument removal (**J**).

**Figure 3:**
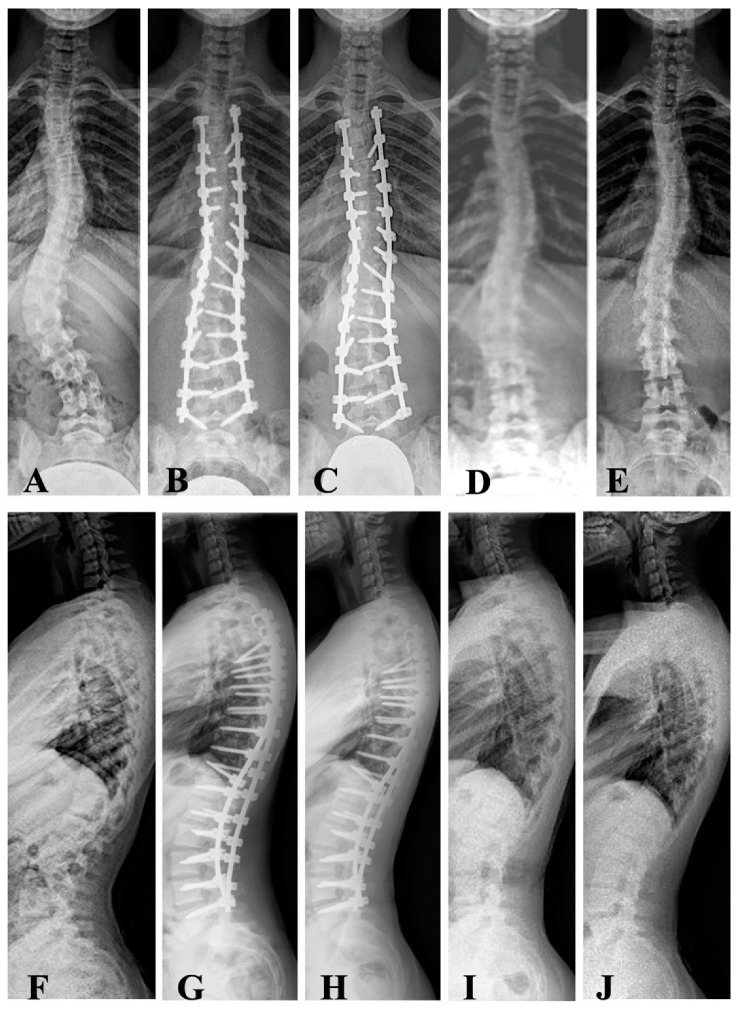
A 14-year-and-11-month-old girl with Lenke type 5 AIS (17/36/57), with T10-L2 kyphosis (+16°) and Risser 3 (**A**,**F**). Posterior pedicle instrumentation T4-L5 (**B**,**G**) was performed, achieving correction of the thoracolumbar and thoracic curve (**B**). In the sagittal plane, the correction of the thoracolumbar transit kyphosis (**G**) was also verified. The coronal and sagittal angular values remained unchanged until the removal of the vertebral instrumentation (**C**,**H**) and in the radiographic study after the removal of the implants (**D**,**I**). In the last radiographic control, two years after the removal of the instrumentation, the thoracic scoliosis (**E**) and the sagittal vertebral angular values remained unchanged (**J**).

Out of the 36 cases included, only two (5.5%) were boys. A total of nine of the 34 girls (26.4%) were still premenarchal. Concerning Lenke criteria [12], 13 curves were classified as type 1A, three type 1B, eight type 1C, one type 2, one type 3, seven type 5s, and three type 6. Of the main thoracic curves, 82% were right-sided. All the left-sided curves were thoracolumbar or lumbar. Table 1 shows the main characteristics of the patients regarding age, anthropometric profile, maturity, and curve particularities for the whole sample and for the two groups of curve types. The sole difference between thoracic and thoracolumbar/lumbar curves was obviously found at the apex level. Table 2 shows the upper and lower instrumented vertebra in the two types of curves. In 10 of the 36 cases, one or two pedicle screws were not instrumented for technical reasons. There were no neurological complications in this series. However, there were two cases of pleural effusion during the first post-op period, and four wound seromas were noted after removal of the instrumentation. The mean follow-up period from instrumentation to removal of the implants was 31.0 ± 5.8 months (range: 22–42) (Figure 2 and Figure 3).

### 3.2. Radiological Outcomes

**Figure 4:**
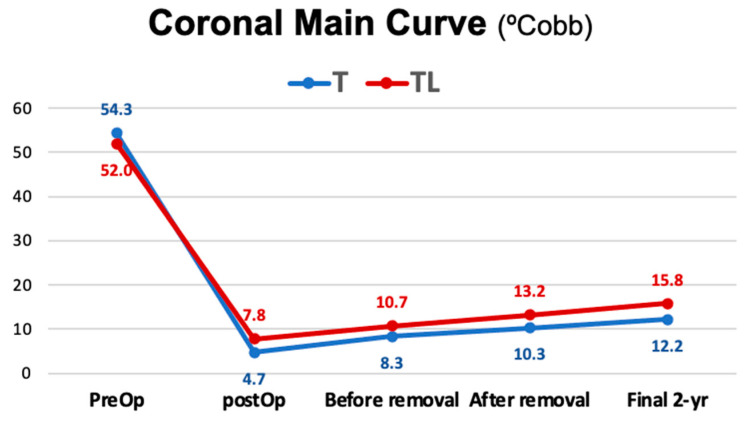
Correction of the main coronal curve during follow-up. A correction of 89.7% was achieved after surgery, and a 75.4% correction was achieved at two years’ follow-up.

Taking into consideration the whole series (Table 3), the analysis of the evolution of the main curve showed a mean preoperative angular value of 53.7° ± 7.50, which changed to a mean angular value of 5.5° ± 3.7° (89.7%) in the immediate postoperative radiograph after surgical treatment. This angular value of scoliosis in the immediate postoperative period after the removal of the instrumentation became an angular value of 8.9° degrees; the value of the final scoliosis was 13.1° at a mean follow-up of 29.8 ± 5.7 months after removal, which represented a 75.4% correction with respect to the initial curve. The results for this parameter were similar in thoracic scoliosis and thoracolumbar and lumbar scoliosis (Figure 4).

**Figure 5:**
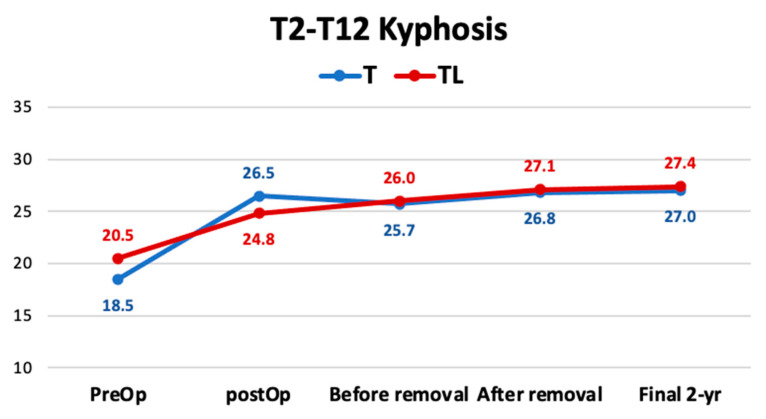
Angular value of the thoracic kyphosis from T2 to T12.

Regarding the lateral plane, the mean preoperative angular value of the thoracic kyphosis from T2 to T12 was 19° and increased to a final angular value of 27.1° in the radiographic study more than two years after the removal of the instrumentation, representing an 29.9% increase in kyphosis. There were no differences in the behavior of thoracic and thoracolumbar curves (Figure 5). Regarding lumbar lordosis, there were no changes before surgery nor two years after the removal of the implants (Table 3).

### 3.3. Sagittal ROM of the Thoracolumbar Transition and Lumbar Spine

**Figure 6:**
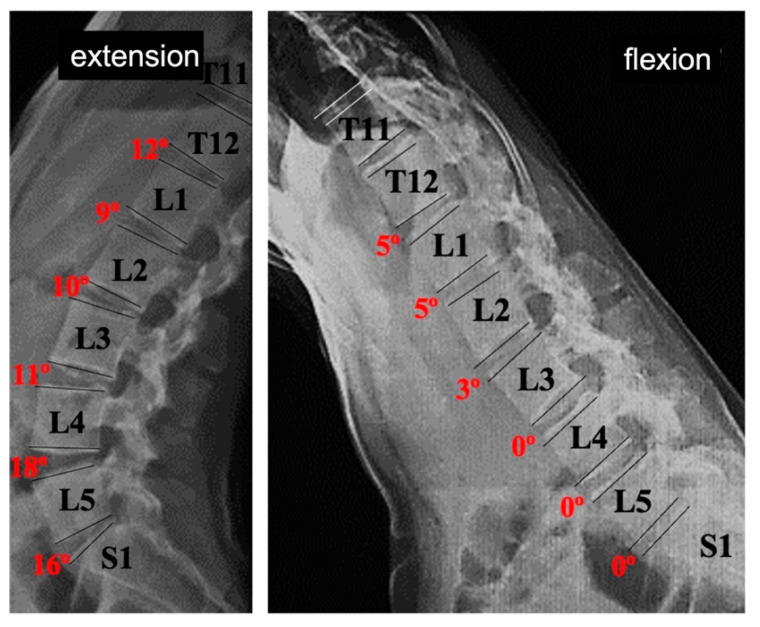
Fourteen-year-old girl with AIS type 1CN Lenke (54° Cobb) who underwent correction surgery with T3–L3 instrumentation. The images show the sagittal X-rays of the lumbar spine in maximal extension and flexion after removal of the implants.

**Figure 7:**
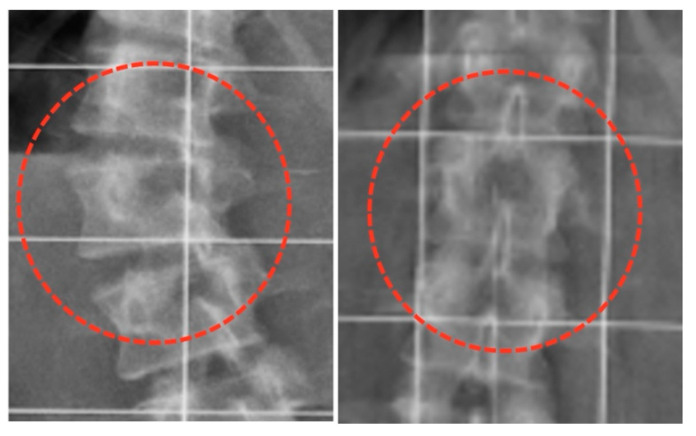
Modulation of the apical vertebra before surgery and after the removal of implants. The wedging ratio increased from 0.75 to 0.96.

Two years after the removal of implants, there was a satisfactory ROM in all analyzed lumbar segments (Figure 6 and Figure 7). The final mean sagittal ROM of the T12-S1 segment was 51.2 ± 21.0°. At the L3-L4 level of the thoracic curves, the average ROM was 28° in addition to that of 13 out of 23 cases; instrumentation at this level was maintained for more than 2 years. In the toracolumbar curves, the average ROM at the L4–L5 level was 27.5°, although five of seven cases were temporarily instrumented. In these curves, the segment with the lower ROM was the L1–L2 that corresponded to the apex level, which is usually the most rigid.

### 3.4. Vertebral Modulation

**Figure 8:**
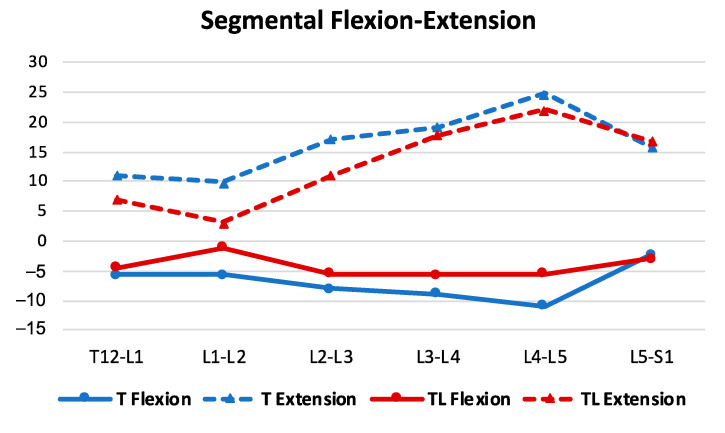
Analysis of thoracolumbar and thoracic ROM during flexion and extension for different segments, two years after implant removal.

The vertebral growth modulation of the epiphyseal plates was analyzed in the vertebral apex according to the WR, which is the result of the height of the vertebral concavity divided by the height of the convexity. The overall results showed that the rectangular shape of the vertebral body of the apex was almost completely restored, with concavity reaching the same height as convexity (Table 4, Figure 8). A greater correction of the thoracolumbar and lumbar curves was found than in thoracic scoliosis. A greater correction was also found in more immature patients.

### 3.5. Quality of Life

**Figure 9:**
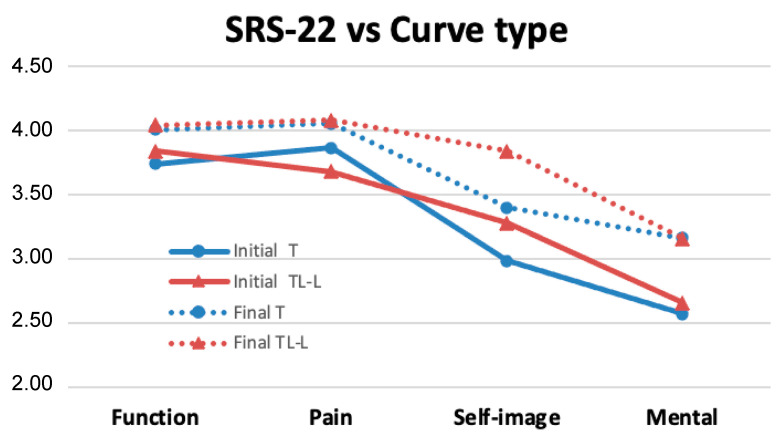
Results of the SRS-22 questionnaire according to curve location. Improvements were demonstrated in many of the tested domains. In most fields of the questionnaire, the thoracic curves improved further than the thoracolumbar curves.

The results of the SRS22 questionnaire demonstrate that this technique produces an improvement in many of the studied domains (Figure 9). For the thoracic curves, the increase in scores was statistically significant in the domains of function (*p* < 0.01), self-image (*p* < 0.01), and mental health (*p* < 0.001) (Table 5), while for the thoracolumbar curves, the improvement was only significant in the mental health domain (*p* < 0.05).

**Figure 10:**
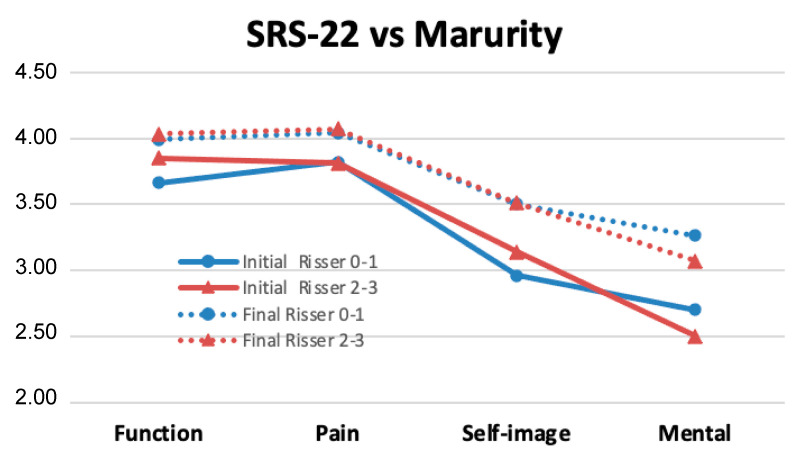
Results of the SRS-22 questionnaire according to the degree of skeletal maturity. A significant improvement was observed in almost all the studied domains.

When the results of the SRS22 questionnaire were stratified according to the degree of skeletal maturity (Figure 10), relevant improvements could also be observed in almost all the domains studied (Table 5). For immature patients (Risser 0–1), the increases in scores were statistically significant in the domains of function (*p* < 0.05), self-image (*p* < 0.05), and mental health (*p* < 0.01). In patients with Risser bone-maturity grade 2–3, the improvement was also significant in these three domains, including function (*p* < 0.05), self-image (*p* < 0.05), and mental health (*p* < 0.01).

At the final follow-up, 33/36 patients (91.6%) stated they were either very satisfied or satisfied. When patients were asked whether they would have the same procedure again, 35/36 patients (97.3%) were definitely or probably sure that they would have this treatment again, and only one was doubtful.

## 4. Discussion

Conventional surgical techniques for the correction of adolescent idiopathic scoliosis involve the fusion of extensive vertebral segments. The definitive immobilization of large vertebral segments increases mechanical stress on adjacent unfused segments, with negative long-term consequences [13]. In addition, these instrumented young patients must live with high-density metallic implants inside their bodies which may condition significant long-term consequences [14,15]. Fusion does not improve preoperative respiratory restriction [16], and it limits the functionality of the thoracolumbar transition and lumbar spine, as some levels of these segments are frequently involved in fusion surgery [17].

Over the past decade, vertebral modulation techniques, both anterior and posterior, have expanded. Anterior vertebral modulation techniques are performed through thoracic approaches, with the significant loss of thoracic continuity and negative consequences on respiratory function [18,19]. These techniques produce unpredictable and frequently insufficient corrections of scoliosis: they do not apply adequate forces bilaterally, nor in a guided manner, and they leave the discs involved in modulation in non-physiological positions, as permanent instrumentation limits disc mobility and compromises future vertebral functionality while also damaging the discs.

The recently published posterior modulation system [10] has been demonstrated to modulate the anteroposterior plane by distraction, using pedicle screws at the ends of the concavity. This system raises certain issues that are related to the minimal number of implants used to correct the three-dimensional deformity; it obtains only partial correction of the anteroposterior plane, and the corrective forces that are applied have a lordotic effect.

We found no previous clinical study reported in the literature describing the restoration of vertebral morphology where the posterior correction of the spinal deformity used only pedicle screws. Studies have not been published to show the effect of implant removal after the complete correction of scoliosis, avoiding the need for an individual to wear metallic implants throughout their entire life. The current study assessed these issues, including the preservation of vertebral motion once the implants were removed.

**Figure 11:**
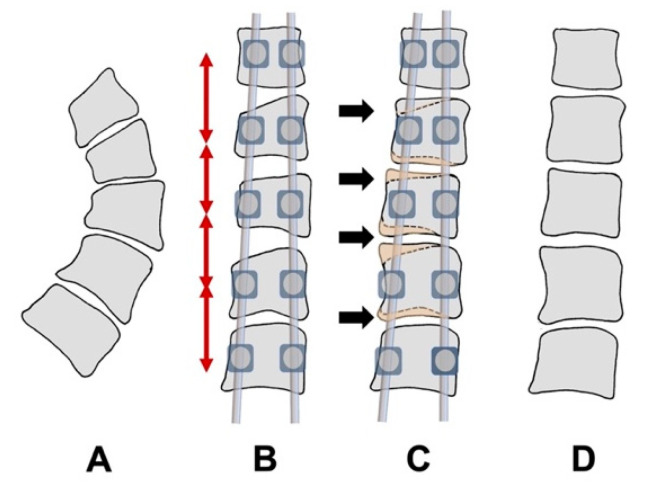
Illustration showing the growth modulation induced by the correction of scoliosis, and posterior stabilization using pedicle screws and rods. (**A**) original curve; (**B**) correction of the major curve with standard techniques using pedicle screws and rods (see red arrows showing distraction forces applied to the intervertebral spaces on the concave side); vertebral wedging was initially maintained at the apical segments; (**C**) the restoration of the apical vertebral shape was achieved by the growth of the epiphyseal plate on the concave side (see arrows) due to distraction of the intervertebral space, while the growth of the convex side was blocked by the lack of distraction made by the pedicle screws on that side; (**D**) the final result after the removal of the instrumentation, showing an almost normal vertebral shape. In the figure, the placement of the transverse hooks at the proximal upper vertebra was not included, because this was not essential for the growth-modulation technique.

In immature patients, conventional posterior vertebral stabilization using all-pedicle screws permitted, by itself, the growth of the epiphyseal plates, particularly at the concave distracted side. In contrast with other techniques, complete correction was achieved initially by leaving the spine in a corrected position and allowing higher growth of the vertebral bodies (Figure 11).

It seems logical to apply a lower density of implants seems in cases of less severe and more flexible scoliosis, where the mechanical stresses required to correct the deformity would be lower. However, the concept should be maintained in order to achieve the complete correction of scoliosis and to apply the correct modulating forces. Bilateral implants close to the physeal plates should preferably be used to enable more effective application of distraction forces at the convex side and to achieve maximum correction, always keeping in mind the necessity to correct and modulate a three-dimensional vertebral deformity. Minor curves should also be instrumented, to avoid their progression and the vertebral imbalances that occur when the main curve is completely corrected [20].

The surgical technique used in the current series was the conventional posterior open approach, because mini-invasive techniques would significantly increase the surgical time and the incidence of pedicle-screw malposition in these patients. This could increase the morbidity associated with this new method, by including a greater number of variables that are difficult to control and so making it difficult to achieve a homogeneous series. Nevertheless, it would be advisable to apply pedicle screws using a mini-invasive or percutaneous approach instead of the conventional open approach, considering that our priority objectives include avoiding damage to muscle and ligament tissue and aiding the recovery of a vertebral range of motion after implant removal [21,22]. In the current study, the lumbar ROM after removal of the implants was similar to that reported in healthy subjects [23].

Regarding the selection of patients included in this study, all cases were adolescent idiopathic scoliosis with more than 45° Cobb for all Lenke types, and this allowed us to observe whether vertebral epiphyseal growth was present in all types of deformity. The limit of bone maturation was a Risser value equal to or less than three, based on a minimum remaining time of growth greater than 18 months so that full correction of the deformity could be effected. The patients included in this study had non-severe scoliosis, and were intentionally selected as more severe scoliosis requires Ponte osteotomies to achieve complete correction of the deformity. Ponte osteotomies are required to enable fusion at the levels where osteotomies are performed, and therefore such cases could not be included in the study.

Removal of the instrumentation was conducted in all cases when patients reached Risser 5 pelvic maturity with complete fusion of the iliac process, to avoid the possibility of increasing residual scoliosis after the removal of the implant. The rationale of this was to avoid the rebound growth observed in other anatomical locations after the removal of guided growth implants [24]. This phenomenon can be prevented by maintaining the implants until bone maturation is complete [24].

The assessment of vertebral epiphyseal growth using the wedging ratio (WR) in the apex vertebra of scoliosis demonstrated the ability of this method to quantify vertebral growth after surgical correction of scoliosis. Although criticism could be raised because the rotation was not assessed, the wedging of vertebrae at the apex could not be explained simply by rotation. An accurate assessment of rotation would have required the repeated use of CT scans, with an unacceptable radiation dosage for the patients involved in the study.

In the current study, the analysis of the flexion–extension mobility of the thoracolumbar transition and lumbar spine was intended to evaluate whether the conventional posterior open approach to the lumbar spine and instrumental immobilization produced a significant limitation of flexion–extension mobility. However, this analysis is complicated because the greatest percentage of lumbar mobility is in the lowest lumbar levels, and these were left unrestricted in most of the cases in this series. This study demonstrated transition from lordosis in extension to kyphosis in flexion, at all lumbar levels in all cases.

From the results obtained in SRS22, it can be inferred that the body image, mental health, pain, and functionality of these patients improved even after two surgical interventions.

The limitation of posterior stabilization without fusion is that it cannot be applied to severe curves of more than 75°, which require Ponte osteotomies for the complete correction of the deformity, implying the fusion of that segment. Therefore, removal of the implants cannot be carried out in these areas. Another limitation is the state of bone maturation of the patients. Those with Risser values higher than three are not suitable for this surgery, because of the limited remaining growth of the epiphyseal plates for the restoration of vertebral body morphology. Furthermore, patients that still have the triradiate cartilage open are also unsuitable for the application of this new technique, because they need a long period until the removal of the instrumentation can be performed. A prolonged period retaining the instrumentation implies a high risk of limitation to the final mobility of the spine, by paraspinal fibrosis or ankylosis of the facet joints.

Posterior stabilization using all-pedicle screws without fusion could probably be extended to less severe curves that have poor prognosis, replacing the use of a brace due to the latter’s limited effectiveness. In these cases, the deformity is more flexible, making the technique less demanding and likely to allow less aggressive instrumentation. In the future, indication for posterior stabilization without fusion could also be extended to infantile and juvenile scoliosis and other curve etiologies in which there are no physeal alterations.

Our goal is to restore normal vertebral morphology and functionality in children suffering from idiopathic scoliosis. With fusionless posterior stabilization, we obtained correction of the deformity that seems comparable to other surgical methods that are currently in use, with the advantage of preserving the range of motion in the whole lumbar spine. The procedure described here does not differ grossly from other fusion techniques that have attempted to achieve correction of spinal deformity using a posterior approach. The main difference of this procedure compared with the traditional fusion technique is that it does not imply fusion: the posterior approach is less traumatic, is performed without injuring the vertebral ligamentous elements, the facet joints are not injured, no bone decortication is conducted, and no bone graft is applied.

It could be argued that fusionless posterior stabilization requires a high density of implants and demands a more exigent correction than conventional posterior fusion, because it requires the instrumentation of a greater number of vertebral levels and a greater number of pedicle screws. In the case of residual deformity, the progression of curves could occur. It should also be stated that the complete correction of scoliosis is challenging for the surgeon, while the surgical technique presented here is similar to posterior fusion techniques.

Although the number of patients in this study was limited, all cases were followed for more than two years after implant removal, and very homogenous results were obtained, giving validity to the study. In addition, the fact that no relapse occurred during follow-up makes it highly unlikely that this will occur at a later stage, particularly after the vertebral body morphology has been restored. Nevertheless, patients remain under periodical review.

## 5. Conclusions

In summary, epiphyseal vertebral growth on the concave side was observed after the correction of scoliotic main curves in AIS patients with Risser 3 bone maturity or less. Curves can be corrected initially through the use of the fusionless posterior approach with conventional all-pedicle screws. Following the restoration of the normal vertebral morphology, the removal of the instrumentation can be performed more than two years after the corrective surgery, once the final bone maturity is reached. Finally, we observed highly satisfactory correction without significant loss of correction during two years of follow-up. This technique permits the conservation of an acceptable ROM in the lower instrumented segments and a final satisfactory correction of spinal deformity.

## Figures and Tables

**Table 1:** Clinical profile and characteristics of the curves.

	Whole Sample *n* = 36 Mean ± SD (95%IC)	Lenke 1,2,3 *n* = 26 Mean ± SD (95%IC)	Lenke 5,6 *n* = 10 Mean ± SD (95%IC)	Z (*p*)
Age (yr.)	13.5 ± 1.3	13.5 ± 2.0	13.5 ± 1.0	0.398 (0.690)
	(13.0–14.0)	(13.1–14.0)	(11.6–15.4)	
Weight (kg)	47.8 ± 9.4	47.3 ± 9.9	49.5 ± 7.6	1.274 (0.203)
	(44.2–51.4)	(42.7–51.8)	(42.4–56.5)	
Stature (cm)	158.1 ± 8.6	158.4 ± 7.6	157.1 ± 11.7	0.345 (0.730)
	(154.7–161.4)	(154.9–161.8)	(146.3–167.9)	
Tanner	2.4 ± 0.8	2.4 ± 0.7	2.3 ± 0.9	0.268 (0.789)
	(2.1–2.7)	(2.1–2.8)	(1.4–3.1)	
Risser	1.2 ± 1.3	1.2 ± 1.6	1.2 ± 1.2	0.028 (0.978)
	(0.7–1.7)	(0.1–2.7)	(0.7–1.8)	
Main curve (Cobb°)	53.7 ± 7.5	54.3 ± 6.7	52.0 ± 10.1	1.595 (0.111)
	(50.7–56.6)	(51.2–57.3)	(42.6–61.3)	
Compensatory curve (Cobb°)	20.4 ± 8.6	18.6 ± 6.6	25.8 ± 11.8	0.051
	(17.1–23.7)	(15.6–21.6)	(14.9–36.7)	
Apex (thoracic level)	9.2 ± 2.9	7.7 ± 1.5	13.7 ± 0.9	4.176 (0.000)
	(8.1–10.4)	(7.1–8.4)	(12.8–14.6)	
Axial rotation (°)	20.4 ± 8.6	18.6 ± 6.6	25.8–11.8	1.569 (0.117)
	(17.1–23.7)	(15.6–21.6)	(14.9–36.7)	
Coronal disequilibrium (mm)	5.8 ± 19.1	2.3 ± 14.5	18.0 ± 28.6	1.832 (0.067)
	(−1.7–13.4)	(−4.9–8.9)	(−11.9–47.9)	
Lateral disequilibrium (mm)	25.6 ± 29.5	30.0 ± 32.9	25.6 ± 29.4	0.234 (0.815)
	(−5.27–56.6)	(15.0–45.0)	(−5.27–56.6)	

**Table 2:** Upper and lower instrumented vertebra in the two types of curves.

	Level	Thoracic (*n* = 26)	Thoracolumbar/Lumbar (*n* = 10)
Superior	T1	1	3
	T2	13	5
	T3	10	-
	T4	2	-
	T6	-	1
	T10	-	1
Inferior	L3	10	-
	L4	16	3
	L5	-	2

**Table 3:** Evolution of coronal and sagittal plane of the main curve along the different stages of follow-up.

	Whole Sample Mean ± SD (95%IC)	Lenke 1, 2, 3 (Thoracic) Mean ± SD (95%IC)	Lenke 5, 6 (Lumbar) Mean ± SD (95%IC)	Z (*p*)
Main Curve (Cobb)				
Initial	53.7 ± 7.5 (50.7–56.6)	54.3 ± 6.7 (51.2–57.3	52.0 ± 10.1 (42.6–61.3)	1.595 (0.111)
Postop	5.5 ± 3.7 (4.1–6.9)	4.7 ± 3.5 (3.1–6.3)	7.8 ± 3.6 (4.5–11.2)	2.232 (0.026)
Before removal	8.9 ± 2.7 (7.1–9.4)	8.3 ± 2.6 (7.1–9.5)	10.7 ± 2.1(8.7–12.7)	1.199 (0.046)
After removal	11.1 ± 2.5 (10.1–12.1)	10.3 ± 2.3 (12.1–11.4)	13.2 ± 1.2 (12.1–14.4)	2.934 (0.003)
Final (2 years post-removal)	13.1 ± 2.9 (11.9–14.2)	12.2 ± 2.8 (10.9–13.4)	15.8 ± 1.3 (14.6–17.1)	3.485 (0.000)
T2-T12 Kyphosis (Cobb)				
Initial	19.0 ± 13.1 (13.8–24.2)	18.5 ± 13.8 (12.3–24.9)	20.5 ± 10.9 (9.0–31.9)	0.554 (0.579)
Postop	26.1 ± 4.7 (22.2–27.9)	26.5 ± 4.7 (24.3–28.7)	24.8 ± 4.8 (20.4–29.3)	0.306 (0.760)
Before removal	25.7 ± 3.3 (24.5–27.1)	25.7 ± 2.6 (24.5–26.9)	26.0 ± 5.2 (21.2–30.8)	0.214 (0.831)
After removal	26.9 ± 3.5 (25.5–28.3)	26.8 ± 3.6 (25.2–28.5)	27.1 ± 3.5 (23.9–30.4)	0.346 (0.729)
Final (2 years post-removal)	27.1 ± 3.8 (11.9–14.2)	27.0 ± 3.8 (25.3–28.8)	27.4 ± 3.6 (24.0–30.8)	0.640 (0.522)
Lumbar lordosis T12-S1(Cobb)				
Initial	55.5 ± 10.3 (51.5–59.6)	56.6 ± 10.8 (51.7–61.5)	51.8 ± 8.1 (43.3–60.4)	0.906 (0.365)
Postop	55.2 ± 7.8 (52.2–58.2)	56.4 ± 8.5 (52.5–60.2)	51.7 ± 3.8 (48.2–55.2)	0.586 (0.558)
Before removal	56.0 ± 6.8 (53.4–58.7)	56.9 ± 7.6 (53.4–60.3)	53.4 ± 3.0 (50.6–56.2)	0.267 (0.790)
After removal	56.7 ± 5.6 (54.5–58.9)	57.4 ± 6.1 (54.6–60.1)	54.7 ± 3.8 (51.2–58.2)	0.748 (0.455)
Final (2 years post-removal)	56.9 ± 6.0 (54.6–59.3)	57.5 ± 6.6 (54.5–60.5)	55.3 ± 3.8 (51.8–58.8)	0.426 (0.670)

**Table 4:** Vertebral modulation analyzed at the vertebral apex using the wedging ratio.

	Initial	Final	Z (*p*)
Whole series	0.70 ± 0.05 (0.67–0.74)	0,98 ± 0.08 (0.94–1.03)	3.408 (0.001)
Type of curve			
Thoracic	0.70 ± 0.07 (0.65–0.75)	0.95 ± 0.05 (0.91–0.99)	2.666 (0.008)
Thoracolumbar/Lumbar	0.70 ± 0.04 (0.66–0.75)	1.03 ± 0.10 (0.92–1.14)	2.201 (0.028)
Skeletal maturity			
Risser 0–1	0.70 ± 0.06 (0.65–0.75)	1.00 ± 0.09 (0.93–1.07)	2.666 (0.008)
Risser 2–3	0.72 ± 0.06 (0.66–0.78)	0.95 ± 0.06 (0.89–1.01)	2.201 (0.028)

**Table 5:** Change in SRS-22 according to the deformity location and skeletal maturity.

	Change in SRS-22			
	Function	Pain	Self-Image	Mental Health
Thoracic				
Mean ± SD	0.26 ± 0.29	0.18 ± 0.37	0.41 ± 0.44	0.58 ± 0.18
Z (*p*)	2.691 (0.007)	1.876 (0.061)	3.025 (0.002)	3.573 (0.000)
Thoracolumbar				
Mean ± SD	0.20 ± 0.31	0.40 ± 0.31	0.56 ± 0.48	0.50 ± 0.17
Z (*p*)	1.289 (0.197)	1.826 (0.068)	1.826 (0.068)	2.060 (0.039)
Risser 0–1				
Mean ± SD	0.33 ± 0.35	0.22 ± 0.34	0.54 ± 0.50	0.36 ± 0.38
Z (*p*)	2.099 (0.036)	1.706 (0.088)	2.530 (0.011)	2.448 (0.014)
Risser 2–3				
Mean ± SD	0.17 ± 0.20	0.25 ± 0.40	0.56 ± 0.25	0.57 ± 0.07
Z (*p*)	2.201 (0.028)	1.841 (0.066)	2.829 (0.005)	2.980 (0.003)

## Data Availability

All the data generated or analyzed during this study are included in this published article.

## References

[1] Jain V., Lykissas M., Trobisch P., Wall E.J., Newton P.O., Sturm P.F., Cahill P.J., Bylski-Austrow D.I. (2014). Surgical aspects of spinal growth modulation in scoliosis correction. Instr. Course. Lect..

[2] Hueter C. (1862). Anatomische studien an den extremitaetengelenken neugeborener und erwachsener. Virchows. Arch. Pathol. Anat. Physiol..

[3] Mehlman C.T., Araghi A., Roy D.R. (1997). Hyphenated history: The Hueter-Volkmann law. Am. J. Orthop..

[4] Stokes I.A., Spence H., Aronsson D.D., Kilmer N. (1996). Mechanical modulation of vertebral body growth. Implications for scoliosis progression. Spine.

[5] Betz R.R., Kim J., D’Andrea L.P., Mulcahey M.J., Balsara R.K., Clements D.H. (2003). An innovative technique of vertebral body stapling for the treatment of patients with adolescent idiopathic scoliosis: A feasibility, safety, and utility study. Spine.

[6] Samdani A.F., Ames R.J., Kimball J.S., Pahys J.M., Grewal H., Pelletier G.J., Betz R.R. (2015). Anterior vertebral body tethering for immature adolescent idiopathic scoliosis: One-year results on the first 32 patients. Eur. Spine J..

[7] Lowe T.G., Wilson L., Chien J.T., Line B.G., Klopp L., Wheeler D., Molz F. (2005). A posterior tether for fusionless modulation of sagittal plane growth in a sheep model. Spine.

[8] Liu J., Li Z., Shen J., Xue X. (2015). Spinal growth modulation with posterior unilateral elastic tether in immature swine model. Spine J..

[9] Zhang H.Y., Li Q.Y., Wu Z.H., Zhao Y., Qiu G.X. (2017). Lumbar Scoliosis Induction in Juvenile Dogs by Three-dimensional Modulation of Spinal Growth Using Nickel-Titanium Coil Springs. Chin. Med. J..

[10] Floman Y., El-Hawary R., Lonner B.S., Betz R.R., Arnin U. (2021). Vertebral growth modulation by posterior dynamic deformity correction device in skeletally immature patients with moderate adolescent idiopathic scoliosis. Spine Deform..

[11] Burgos J., Barrios C., Hevia E., Antón-Rodrigálvarez L.M. Abolition of Sagittal T7-T10 Dynamics During Forced Ventilation in Lenke 1A AIS Patients as Compared to Healthy Subjects. An Underlying Factor of the Respiratory Limitation in AIS. Proceedings of the 53rd SRS Annual Meeting & Course.

[12] Lenke L.G., Betz R.R., Harms J., Bridwell K.H., Clements D.H., Lowe T.G., Blanke K. (2001). Adolescent idiopathic scoliosis: A new classification to determine extent of spinal arthrodesis. J. Bone. Joint. Surg. Am..

[13] Kepler C.K., Meredith D.S., Green D.W., Widmann R.F. (2012). Long-term outcomes after posterior spine fusion for adolescent idiopathic scoliosis. Opin. Pediatr..

[14] del Rio J., Beguiristain J., Duart J. (2007). Metal levels in corrosion of spinal implants. Eur. Spine J..

[15] Mathew S.E., Xie Y., Bagheri L., Claton L.E., Chu L., Badreldin A., Abdel M.P., van Wijnen A.J., Haft G.F., Milbrandt T.A. (2022). Serum Ion Levels Elevated in Pediatric Patients With Metal Implants?. J. Pediatr. Orthop..

[16] Lorente A., Barrios C., Burgos J., Hevia E., Fernández-Pineda L., Lorente R., Rosa B., Pérez-Encinas C. (2017). Cardiorespiratory Function Does Not Improve 2 Years After Posterior Surgical Correction of Adolescent Idiopathic Scoliosis. Spine.

[17] Burgos J., Barrios C., Mariscal G., Lorente A., Lorente R. (2021). Non-uniform Segmental Range of Motion of the Thoracic Spine During Maximal Inspiration and Exhalation in Healthy Subjects. Front. Med..

[18] Namboothiri S., Kumar R., Menon K.V. (2005). Early changes in pulmonary function following thoracotomy for scoliosis correction: The effect of size of incision. Eur. Spine J..

[19] Lonner B.S., Auerbach J.D., Estreicher M.B., Betz R.R., Crawford A.H., Lenke L.G., Newton P.O. (2009). Pulmonary function changes after various anterior approaches in the treatment of adolescent idiopathic scoliosis. J. Spinal. Disord. Tech..

[20] Ritzman T.F., Upasani V.V., Bastrom T.P., Betz R.R., Lonner B.S., Newton P.O. (2008). Comparison of compensatory curve spontaneous derotation after selective thoracic or lumbar fusions in adolescent idiopathic scoliosis. Spine.

[21] Sarwahi V., Horn J.J., Kulkarni P.M., Wollowick A.L., Lo Y., Gambassi M., Amaral T.D. (2016). Minimally Invasive Surgery in Patients With Adolescent Idiopathic Scoliosis: Is it Better than the Standard Approach? A 2-Year Follow-up Study. Clin. Spine Surg..

[22] Si G., Li T., Wang Y., Liu X., Li C., Yu M. (2021). Minimally invasive surgery versus standard posterior approach for Lenke Type 1–4 adolescent idiopathic scoliosis: A multicenter, retrospective study. Eur. Spine J..

[23] Yukawa Y., Matsumoto T., Kollor H., Minamide A., Hashizume H., Yamada H., Kato F. (2019). Local Sagittal Alignment of the Lumbar Spine and Range of Motion in 627 Asymptomatic Subjects: Age-Related Changes and Sex-Based Differences. Asian Spine J..

[24] Choi K.J., Lee S., Park M.S., Sung K.H. (2022). Rebound phenomenon and its risk factors after hemiepiphysiodesis using tension band plate in children with coronal angular deformity. BMC Musculoskelet. Disord..

